# *SRSF1* haploinsufficiency is responsible for a syndromic developmental disorder associated with intellectual disability

**DOI:** 10.1016/j.ajhg.2023.03.016

**Published:** 2023-04-17

**Authors:** Elke Bogaert, Aurore Garde, Thierry Gautier, Kathleen Rooney, Yannis Duffourd, Pontus LeBlanc, Emma van Reempts, Frederic Tran Mau-Them, Ingrid M. Wentzensen, Kit Sing Au, Kate Richardson, Hope Northrup, Vincent Gatinois, David Geneviève, Raymond J. Louie, Michael J. Lyons, Lone Walentin Laulund, Charlotte Brasch-Andersen, Trine Maxel Juul, Fatima El It, Nathalie Marle, Patrick Callier, Raissa Relator, Sadegheh Haghshenas, Haley McConkey, Jennifer Kerkhof, Claudia Cesario, Antonio Novelli, Nicola Brunetti-Pierri, Michele Pinelli, Perrine Pennamen, Sophie Naudion, Marine Legendre, Cécile Courdier, Aurelien Trimouille, Martine Doco Fenzy, Lynn Pais, Alison Yeung, Kimberly Nugent, Elizabeth R. Roeder, Tadahiro Mitani, Jennifer E. Posey, Daniel Calame, Hagith Yonath, Jill A. Rosenfeld, Luciana Musante, Flavio Faletra, Francesca Montanari, Giovanna Sartor, Alessandra Vancini, Marco Seri, Claude Besmond, Karine Poirier, Laurence Hubert, Dimitri Hemelsoet, Arnold Munnich, James R. Lupski, Christophe Philippe, Christel Thauvin-Robinet, Laurence Faivre, Bekim Sadikovic, Jérôme Govin, Bart Dermaut, Antonio Vitobello

**Affiliations:** 1 Center for Medical Genetics, Ghent University Hospital, 9000 Ghent, Belgium; 2 Department of Biomolecular Medicine, Faculty of Medicine and Health Sciences, Ghent University, 9000 Ghent, Belgium; 3 UMR1231 GAD, Inserm - Université de Bourgogne, Dijon, France; 4 Centre de Référence Maladies Rares "Anomalies du Développement et Syndromes Malformatifs", Centre de Génétique, FHU-TRANSLAD, CHU Dijon Bourgogne, 21000 Dijon, France; 5 University Grenoble Alpes, Inserm U1209, CNRS UMR 5309, Institute for Advanced Biosciences (IAB), 38000 Grenoble, France; 6 Department of Pathology and Laboratory Medicine, Western University, London, ON N5A 3K7, Canada; 7 Verspeeten Clinical Genome Centre, London Health Science Centre, London, ON N6A 5W9, Canada; 8 Unité Fonctionnelle Innovation en Diagnostic génomique des maladies rares, FHU-TRANSLAD, CHU Dijon Bourgogne, 21000 Dijon, France; 9 GeneDx, Gaithersburg, MD, USA; 10 Division of Medical Genetics, Department of Pediatrics, McGovern Medical School at the University of Texas Health Science Center at Houston (UTHealth Houston), Houston, TX, USA; 11 Children’s Memorial Hermann Hospital, Houston, TX, USA; 12 Unité de Génétique Chromosomique, CHU Montpellier, Montpellier, France; 13 Montpellier University, Inserm U1183, Montpellier, France; 14 Reference center for rare disease developmental anomaly malformative syndrome, Department of Medical Genetics, Montpellier Hospital, Montpellier, France; 15 Greenwood Genetic Center, Greenwood, SC, USA; 16 Department of Paediatrics, Odense University Hospital, Odense, Denmark; 17 Department of Clinical Genetics, Odense University Hospital, 5000 Odense, Denmark; 18 Human Genetics, Department of Clinical Research, Health Faculty, University of Southern Denmark, 5000 Odense, Denmark; 19 Laboratoire de Génétique Chromosomique et Moléculaire, Pôle de Biologie, CHU de Dijon, Dijon, France; 20 Translational Cytogenomics Research Unit, Bambino Gesù Children’s Hospital, IRCCS, Rome, Italy; 21 Telethon Institute of Genetics and Medicine, Pozzuoli, Italy; 22 Department of Translational Medicine, University of Naples Federico II, Naples, Italy; 23 Medical Genetics Department, CHU Bordeaux, Bordeaux, France; 24 INSERM U1211, Laboratoire MRGM, Bordeaux University, Bordeaux, France; 25 Pathology Department, CHU Bordeaux, Bordeaux, France; 26 Service de génétique, CHU de Reims, Reims, France; 27 Service de génétique médicale, CHU de Nantes, Nantes, France; 28 L’institut du thorax, INSERM, CNRS, UNIV Nantes, CHU de Nantes, Nantes, France; 29 Broad Center for Mendelian Genomics, Program in Medical and Population Genetics, Broad Institute of MIT and Harvard, Cambridge, MA, USA; 30 Victorian Clinical Genetics Services, Murdoch Children’s Research Institute, Parkville, VIC, Australia; 31 Department of Pediatrics, Baylor College of Medicine, San Antonio, TX, USA; 32 Department of Molecular and Human Genetics, Baylor College of Medicine, Houston, TX, USA; 33 Section of Pediatric Neurology and Developmental Neuroscience, Department of Pediatrics, Baylor College of Medicine, Houston, TX, USA; 34 Texas Children’s Hospital, Houston, TX, USA; 35 Internal Medicine A, Danek Gertner Institute of Human Genetics, Sheba Medical Center, Ramat Gan, Israel; 36 Sackler Faculty of Medicine, Tel Aviv University, Tel Aviv, Israel; 37 Baylor Genetics Laboratories, Houston, TX, USA; 38 Institute for Maternal and Child Health, IRCCS Burlo Garofolo, Trieste, Italy; 39 UO Genetica Medica, IRCCS Azienda Ospedaliero-Universitaria di Bologna, Bologna, Italy; 40 Neonatal Intensive Care Unit, Maggiore Hospital, Bologna, Italy; 41 Department of Medical and Surgical Sciences, University of Bologna, Bologna, Italy; 42 Université Paris Cité, Imagine Institute, INSERM UMR1163, Paris 75015, France; 43 Department of Neurology, Ghent University Hospital, 9000 Ghent, Belgium; 44 Human Genome Sequencing Center, Baylor College of Medicine, Houston, TX, USA; 45 Centre de Référence Maladies Rares « Déficiences intellectuelles de causes rares », Centre de Génétique, FHU-TRANSLAD, CHU Dijon Bourgogne, Dijon, France

**Keywords:** SRSF1, splicing, neurodevelopmental disorder, haploinsufficiency, Drosophila, epigenetic signature

## Abstract

SRSF1 (also known as ASF/SF2) is a non-small nuclear ribonucleoprotein (non-snRNP) that belongs to the arginine/serine (R/S) domain family. It recognizes and binds to mRNA, regulating both constitutive and alternative splicing. The complete loss of this proto-oncogene in mice is embryonically lethal. Through international data sharing, we identified 17 individuals (10 females and 7 males) with a neurodevelopmental disorder (NDD) with heterozygous germline *SRSF1* variants, mostly *de novo*, including three frameshift variants, three nonsense variants, seven missense variants, and two microdeletions within region 17q22 encompassing *SRSF1*. Only in one family, the *de novo* origin could not be established. All individuals featured a recurrent phenotype including developmental delay and intellectual disability (DD/ID), hypotonia, neurobehavioral problems, with variable skeletal (66.7%) and cardiac (46%) anomalies. To investigate the functional consequences of *SRSF1* variants, we performed *in silico* structural modeling, developed an *in vivo* splicing assay in *Drosophila*, and carried out episignature analysis in blood-derived DNA from affected individuals. We found that all loss-of-function and 5 out of 7 missense variants were pathogenic, leading to a loss of SRSF1 splicing activity in *Drosophila*, correlating with a detectable and specific DNA methylation episignature. In addition, our orthogonal *in silico*, *in vivo*, and epigenetics analyses enabled the separation of clearly pathogenic missense variants from those with uncertain significance. Overall, these results indicated that haploinsufficiency of *SRSF1* is responsible for a syndromic NDD with ID due to a partial loss of SRSF1-mediated splicing activity.

## Teaser

Thanks to a large international data-sharing effort, *in silico* structural protein modeling, DNA methylation episignature analyses, and *in vivo* splicing assays in *Drosophila*, Bogaert et al. demonstrate that haploinsufficiency of *SRSF1*, which encodes a pre-mRNA splicing factor, causes a syndromic neurodevelopmental disorder with mild to moderate intellectual disability.

## Introduction

Neurodevelopmental disorders (NDDs) affect around 3% of individuals worldwide and have an incidence of approximately 2%–5% of births.^1^ They are characterized by abnormal cognitive functioning, which may affect behavior, learning, thinking, reasoning, remembering, problem-solving, decision-making, and attention. Intellectual disability (ID), autism spectrum disorder (ASD), attention-deficit/hyperactivity disorder (ADHD), schizophrenia, and bipolar disorders lie on a neurodevelopmental continuum.^2^ NDDs are caused by numerous etiologies including environmental and genetic factors, resulting in a heterogeneous group of diseases with possible clinical overlap. Genetic causes, and in particular *de novo* mutations, likely account for more than 80% of individuals affected by NDDs, with an increasing number of contributing genes being recognized worldwide.^3^^,^^4^^,^^5^

The implementation of next-generation sequencing (NGS) in clinical practice has allowed the identification of variants in “novel” genes or the implication of known disease-associated genes in new phenotypes. Clinical exome sequencing (ES) targeting genes involved in human genetic disorders (OMIM-morbid genes) has become a first-tier genetic tool to identify the potential genetic contributory cause of NDD and to reduce the diagnostic odyssey. ES can also be extended to non-OMIM-morbid and non-OMIM genes that, after recurrence by data sharing and genotype-phenotype correlation studies, can be identified as a genetic cause of newly described human disorders.^6^^,^^7^ However, this “conventional” manner of testing still leaves a substantial portion of affected individuals genetically undiagnosed or with genetic variants of uncertain clinical significance (VUSs). This is especially the case in diseases with high phenotypic and genetic heterogeneity, such as NDDs. Recently, advances in epigenetics have provided a complementary approach for VUS assessment and reclassification, through the analysis of genome-wide DNA methylation patterns associated with a single gene or a group of genes belonging to the same pathway.^8^^,^^9^^,^^10^^,^^11^^,^^12^^,^^13^^,^^14^^,^^15^

*SRSF1* (MIM: 600812) is located in the 17q22 region and encodes a polyfunctional protein regulating a diverse set of cellular processes all related to the information flow from DNA to RNA to protein. Maintenance of genomic stability, transcriptional regulation, mRNA nuclear export, mRNA stability and quality control, nonsense-mediated mRNA decay (NMD), and translation are all processes in which SRSF1 plays a role.^16^^,^^17^^,^^18^^,^^19^^,^^20^ However, SRSF1 is best known for its role in constitutive and alternative pre-mRNA splicing.^21^ All three protein domains, the two RNA recognition motifs (RRMs) and the R/S domain, cooperate to regulate the splicing actions of SRSF1. The C-terminally located R/S domain promotes splicing by attracting limiting splicing factors to the pre-mRNA. This domain interacts with 5′- and 3′-splicing components and the branchpoint sequence to bridge 5′-and 3′-splice sites (SS).^17^^,^^19^^,^^22^^,^^23^ Phosphorylation of serine residues in this domain acts as a molecular switch to regulate and coordinate the actions of the R/S domain with those of the RRMs. The hypo-phosphorylated R/S domain interacts preferentially intramolecularly with its own RRM domains, whereas the hyper-phosphorylated R/S domain facilitates the recruitment of SRSF1 to active sites of transcription, where the RRMs can bind preferentially to exonic splicing enhancer (ESE) sequences.^24^ Upon ESE binding, the RRMs consolidate U1 snRNP, binding to a 5′-SS-containing pre-mRNA by interacting with U1 70K and/or with U1 snRNA, both specific components of U1 snRNP.^23^^,^^24^ The interaction with U1 70K is mediated by the first RRM domain (RRM1) simultaneously binding the ESE site and U1 70K.^24^ For the interaction with the U1 snRNA, both RRMs are involved and bind stem loop 3 of the U1 snRNA.^23^ Both SRSF1 and U1 snRNP components stimulate exon inclusion and affect 5′-SS selection.^19^^,^^23^ Besides splicing enhancing, SRSF1 promotes exon skipping events as well. This function is thought to reside within the second RRM (RRM2, also referred to as pseudo RRM). The pseudo RRM regulates splicing by competing with the binding of other splicing factors to a GGA motif in the pre-mRNA rather than by recruiting them to the cassette exon.^25^ SRSF1 is known to activate or repress the inclusion of hundreds of exons, and this activity is thought to be the main reason why it is an essential protein.^26^^,^^27^^,^^28^ In mice, the complete loss of SRSF1 is embryonically lethal, highlighting an important role for SRSF1 during development.^29^ Not surprisingly, a tight control on *SRSF1* expression levels is crucial for cellular survival, and several feedback loops are at play to monitor its levels. A first mechanism is exerted by alternative splicing of its own pre-mRNA. These alternative transcripts contain premature termination codons (PTCs), which are targets for NMD. Shifting the translation mode from polysomes to monosomes, hence reducing translation efficiency for its own mRNA, is a second mechanism of autoregulation. Finally, SRSF1 modulates the expression of regulatory miRNAs.^19^ Misregulation of SRSF1 levels is linked to diseases. Its overexpression, leading to dysregulation of alternative splicing (AS), has been reported in several human tumors including lung, colon, pancreas, and breast tumors.^30^^,^^31^^,^^32^ Somatic variants in *SRSF1* had also been described in hematologic malignancies to be critical for the regulation of gene expression leading to leukemogenesis.^33^ However, the effects of *SRSF1* pathogenic germline variants have not been reported.

In this work, through international data sharing, we describe clinical and molecular data from 17 individuals with heterozygous germline variations in *SRSF1* and a syndromic form of developmental delay. We provide *in silico*, *in vivo*, and *in vitro* functional evidence in support of the pathogenicity of *SRSF1* haploinsufficiency.

## Material and methods

### Research subjects

All affected individuals or their legal representative gave their informed consent for the sequencing procedures and the publication of their results along with clinical and molecular data. Special consent forms were signed authorizing publication of pictures when relevant. The study was performed within the framework of the GAD (“Génétique des Anomalies du Développement”) collection and approved by the appropriate institutional review board of Dijon University Hospital (DC2011-1332).

### Genetic analyses

Solo or trio ES variant filtering and analysis were performed in individuals 1–5 and 7–16 at the respective institutions (supplemental methods). Alignment was made on the reference human genome GRCh37/hg19. Array-comparative genomic hybridization analysis (aCGH) was performed for individual 6 with a 44K whole-genome microarray (Agilent Technologies, Santa Clara, CA, USA) according to the manufacturer’s instructions. Confirmation and segregation analysis of single-nucleotide or indel variants as well as the 17q22 microdeletion were performed by Sanger sequencing or quantitative polymerase chain reaction (PCR), respectively. We used the RefSeq (GenBank: NM_006924.5) transcript as reference. In individual 17, the deletion was confirmed by FISH (RP11-102J6); parents were negative by FISH. The array data came from Baylor College of Medicine Medical Genetics Laboratory (v.8.1 oligo microarray, 180K custom array from Agilent). The v.8.1 oligonucleotide microarray contains 180,000 oligonucleotides with exon-level coverage for over 1,700 genes (average of 4.2 probes/exon). The manufacturing of these arrays and microarray procedures has been previously described.^34^

### Protein structural analysis

*Drosophila* and human SRSF1 predicted structures were obtained from the AlphaFold Protein Structure Database. Files were loaded and aligned in PyMol (v.2.52). Flexible portions of the human sequence were trimmed to restrict information around RRM1 and RRM2. Data were validated by comparison with structures of both RRMs stored in the Protein Data Bank (PDB). In PyMol, both RRMs were rendered as a cartoon, and amino acids involved in the different alterations were added as spheres along the cartoon structure. When required, surface rendering was used with a transparency set to 1.0 to render a solid appearance or with a transparency set to 0.3 to allow for transparency through the structure. Involved amino acids were modeled as a colored side chain on the corresponding RRM and illustrated as spheres. A simulated model of the mutated amino acid was generated with the Wizard mutagenesis module in PyMol and rendered as described above. The wild-type (WT) and mutated situations were oriented and rendered to exhibit the surface modifications generated by the exchange of amino acid. The RRM sequence carrying the alteration was rendered as a semi-transparent surface set to 0.3, while the other one was rendered as a less semi-transparent surface (0.6). All chains were colored with a 70% gray level in PyMol.

### Fly stocks and maintenance

*Drosophila* strains were maintained on standard Nutrifly formula food, yellow cornmeal, agar (type II), corn syrup solids, inactive nutritional yeast, and soy flour (Genesee Scientific) in a 12 h light/dark rhythm temperature-controlled environment. The w^1118^ (Canton-S10) line and luciferase line (Bloomington Drosophila Stock Center, y[1]v[1]; P{y[+t7.7] v[+t1.8] = UAS-LUC.VALIUM10}attP2, BDSC#35788) were used as control, and the UAS-RFP line (w[^∗^]; P{w[+mC] = UAS-mCD8.ChRFP}3, BDSC#27392), UAS-RFP-NLS line (w[^∗^]; P{w[+mC] = UAS-mCherry.NLS}3, BDSC#38424), GMR-GAL4 line (Flystock, P. Callaerts, KU Leuven), CCAP-GAL4 line (y[1] w[^∗^]; P{w[+mC] = CCAP-GAL4.P}16/CyO, BDSC#25685), and Nsyb-GAL4 line (y[1] w[^∗^]; P{w[+m^∗^] = nSyb-GAL4.S}3, BDSC#51635) were used to drive expression in the fly eye or pan-neuronally. To generate WT and mutant UAS-SRSF1 and UAS-SF2 fly lines, the coding region of those genes was subcloned in the pUAST-attB backbone (GenScript Biotech, the Netherlands), allowing the generation of transgenic fly lines by targeted insertion into the 68A4 attP locus on the third chromosome (GenetiVision, USA). We generated these UAS lines with and without the N-terminal GFP tag. Crosses for adult offspring frequencies and phenotypic data were performed at 25° for fly proteins and 29°C for human proteins.

### *Drosophila* AS analysis

Thirty brains from third-instar larvae expressing SRSF1 and SF2 under control of the GMR enhancer were dissected, and RNA was isolated using a Quick-RNA Tissue/Insect kit (Zymo Research, CA, USA). The RNA samples were sent for RNA sequencing (Macrogen Europe). The library preparation was done with an Illumina TruSeq Stranded Total RNA Ribo-Zero Gold kit, and the sequencing was done on a NovaSeq 6000 sequencer. Samples were run on an S4 flow cell at 50M reads/sample. The resulting Fasta files were aligned using TopHat v.2.2.1. Cufflinks v.2.2.1 was used to generate QC data and count files for the downstream analysis. For analyzing AS patterns, the rMATS turbo v.4.1.1 computational tool was used.^35^ rMATS detects the five primary types of splicing events: alternative 3′-splice sites (A3SSs), alternative 5′ splice sites (A5SSs), skipped exons (SEs), retained introns (RIs), and mutually exclusive exons (MXEs). Additionally, it computes the p value and false discovery rate (FDR) of the ratio of isoforms between the two study conditions filtered by a user-defined difference threshold. For our analysis the threshold was left at the default setting of 0.0001 (0.01% splicing difference). AS events were selected as significant when the conditions FDR ≤ 0.01 and |Ψ| ≥ 0.1 were met.

### *Drosophila* misexpression studies: Offspring quantification and external eye phenotype

#### Offspring assay

For each *Drosophila* cross, the collected offspring were divided by sex, and the genotypes were counted according to the balancers. The offspring ratio was determined by dividing counted offspring by expected offspring.

#### External eye phenotype quantifications

Adult flies were anesthetized with CO_2_, and images were taken with a zoom stereo microscope (Leica Z16 APO). The irregularity score was calculated by the Fiji plugin FLEYE, designed by Diez-Hermano and collaborators.^36^ The pigmentation score was calculated using the color histogram tool in ImageJ. The percentage of red pixels in the fly eye was measured. In each condition a minimum of 10 female and 10 male flies were analyzed. A Kruskal-Wallis test with multiple comparison analysis was used to process the data statistically.

### *Drosophila* immunohistochemistry

Third-instar larval brains were dissected, fixed for 20 min in 4% PFA, and mounted using Fluoromount-G Mounting Medium. UAS lines expressing GFP-tagged SRSF1 variants alongside RFP or RFP-NLS were expressed in bursicon neurons using a Crustacean cardioactive peptide CCAP-GAL4 driver line.

### DNA methylation episignature analysis

The DNA methylation study was approved by the Western University Research Ethics Board (REB 106302, 10 August 2020). The analysis was performed with 500 ng of bisulfite-converted DNA as the input, using the Illumina Infinium MethylationEPIC BeadChip arrays (EPIC array) according to the manufacturer’s protocols (Illumina, San Diego, CA, USA). Analysis and discovery of episignatures were carried out based on our laboratory’s previously published protocols.^37^ In brief, intensity data files (IDATs) containing the methylated and unmethylated signal intensities were analyzed in R v.4.1.1. Methylation data normalization was performed using the Illumina method, with background correction using the R minfi package v.1.40.0.^38^ Probes with detection p value > 0.01, probes located on chromosomes X and Y, probes containing single-nucleotide polymorphisms (SNPs) at or near the CpG interrogation site or single-nucleotide extension site, and probes that cross-react with other genomic regions were eliminated. Samples containing failed probes of >5% (p value >0.1, calculated by the R minfi package v.1.40.0) were removed. Principal-component analysis (PCA) was performed to examine batch structure and identify outliers. Matched controls, at a ratio of 1:5, were randomly selected from the EpiSign Knowledge Database (EKD)^9^ and matched by array type, sex, and age using the R MatchIt package v.4.3.4.^39^ Methylation levels for each probe (beta values) were converted to M values by logit transformation, and linear regression was applied to identify differentially methylated probes (DMPs) using the R limma package v.3.50.0.^40^ Estimated blood cell proportions were incorporated into the model matrix as confounding variables.^41^ p values were moderated using the eBayes function in the R limma package v.3.50.0.^40^

Episignature probe selection was performed in three stages. First, 800 probes were retained with the highest product of methylation differences between *SRSF1* samples and controls and the negative of the logarithm of p values. Next, a receiver’s operating characteristic (ROC) curve analysis was performed, and 267 probes were retained with the highest area under the curve. Lastly, probes with pairwise correlation greater than 0.6 measured using Pearson’s correlation coefficients for all probes were eliminated. Unsupervised clustering models were applied, using the remaining 107 episignature probes, including hierarchical clustering using Ward’s method on Euclidean distance in the R gplots package v.3.1.1 (https://CRAN.R-project.org/package=gplots) and multidimensional scaling (MDS) by scaling of the pairwise Euclidean distances between samples. The robustness of the episignature was assessed using multiple rounds of “leave one out” cross-validation: in each round, one sample was used for testing, and the remaining samples were used for probe selection. The corresponding unsupervised clustering plots were visualized. A support vector machine classifier (SVM) was trained using the R e1071 package v.1.7–9 and to construct a multi-class prediction model as previously described.^9^

### Functional correlation of the genome-wide methylation profiles of *SRSF1* and EpiSign disorders

Functional annotation and EpiSign cohort comparisons were performed according to our previously published methods.^37^ In brief, to establish the genome-wide methylation profile of the *SRSF1* cohort, we used the same nine *SRSF1* samples as training and matched to array-, age-, and sex-matched controls at the same 1:5 ratio. Controls for correlation analysis consisted of samples from unaffected individuals as well as individuals negative for other episignatures in the EKD. In order to perform comparison with the previously published EpiSign disorders, only probes present in both the EPIC array and its predecessor, the Illumina 450K array (Illumina, San Diego, CA, USA), were considered for selection. Only probes with a methylation difference >5% and adjusted p value <0.01 were retained. To assess the percentage of DMPs shared between the *SRSF1* episignature and the 56 other neurodevelopmental conditions on the EpiSign v.3 clinical classifier, heatmaps and circos plots were produced. Heatmaps were generated using the R package pheatmap (v.1.0.12) and circos plots using the R package circlize (v.0.4.15).^42^ In order to assess the relationship between the *SRSF1* cohort and the 56 other EpiSign disorders, the distance and similarities between cohorts were analyzed using clustering methods and visualized on a tree-and-leaf plot. This assessed the top 500 DMPs for each cohort, ranked by p value. For cohorts with less than 500 DMPs, all DMPs were used. Tree-and-leaf plots were generated using the R package TreeAndLeaf (v.1.6.1) (https://www.bioconductor.org, accessed October 2022), showing additional information including global mean methylation difference and total number of DMPs identified for each cohort.

### Identification of differentially methylated regions (DMRs)

DMRs were then detected, based on the list of DMPs produced for functional correlation above, using the DMRcate package in R (v.2.8.3),^43^ and regions containing at least five significantly different CpGs within 1,000 bp, with a minimum absolute mean methylation difference between *SRSF1* samples and controls of 0.05 and significant results were chosen using a Fisher’s multiple comparison p value cut-off of <0.01. DMRs were annotated using the UCSC Genome Browser Data Integrator with GENCODE v.3lift37 comprehensive annotations and further characterized using UCSC Genome Browser tools (https://genome.ucsc.edu).

### Annotation of DMPs and DMRs

To determine the genomic location of the DMPs and DMRs, probes were annotated in relation to CpG islands (CGIs) and genes using the R package annotatr (v.1.20.0)^44^ with AnnotationHub (v.3.2.2) and annotations hg19_cpgs, hg19_basicgenes, hg19_genes_intergenic, and hg19_genes_intronexonboundaries. CGI annotations included CGI shores from 0 to 2 kb on either side of CGIs, CGI shelves from 2 to 4 kb on either side of CGIs, and inter-CGI regions encompassing all remaining regions. For gene annotations, “promoter” included up to 1 kb upstream of the transcription start site (TSS) and “promoter+” included the region 1–5 kb upstream of the TSS. Annotations to untranslated regions (5′ UTR and 3′ UTR), exons, introns, and exon/intron boundaries were combined into the “gene body” category.

## Results

### *SRSF1* variants are common genetic defects in a cohort of 17 individuals with neurodevelopmental disorders

**Figure 1:**
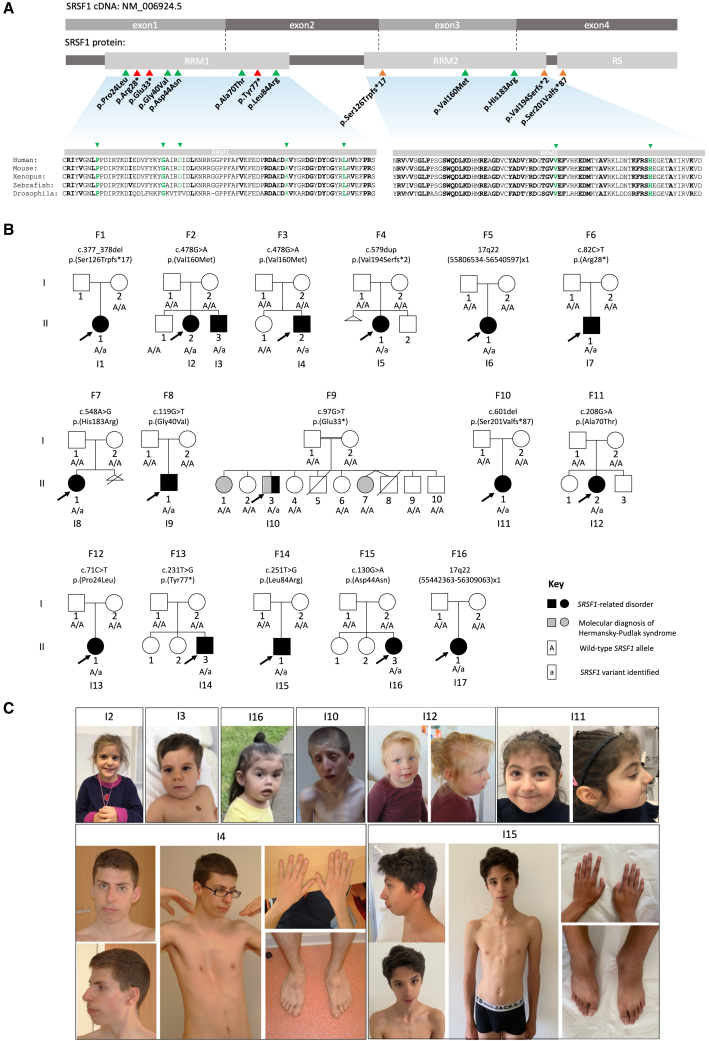
Clinical variants in *SRSF1* cause syndromic developmental disorder associated with intellectual disability (A) Gene, transcript, and protein structure of SRSF1. Clinical variants were shown on the protein level. Evolutionary conservation of the RRM domains is shown with bold amino acids showing evolutionarily conserved residues. Missense variants are indicated by green arrows, nonsense variants are indicated in red, and frameshift variants in orange. (B) Pedigrees of the 16 families reported in this cohort. (C) Photographs of individuals with *SRSF1* variants. Nonspecific facial features were observed in the individuals. Individuals 4 and 15 were referred for marfanoid features: they presented dolichostenomelia, arachnodactyly, and pectus deformity.

Singleton ES was performed in individual 1 (I1 [F1-II-1], Figure 1) referred for NDD associated with facial dysmorphism and skeletal features. The aCGH and ES analysis failed to detect pathogenic variants in OMIM-morbid genes. Research reanalysis identified a heterozygous frameshift variant, c.377_378del (GenBank: NM_006924.5) (p.Ser126Trpfs^∗^17) (Table 1) in *SRSF1*, absent in the gnomAD database. This gene is intolerant to loss-of-function (LoF) variant alleles with an associated probability of LoF intolerance (pLI) score of 0.98 (gnomAD v.2.1.1, https://gnomad.broadinstitute.org), a LoF observed/expected upper bound fraction (LOEUF) score of 0.24, and a haploinsufficiency (HI) index score of 1.47 by DECIPHER (https://www.deciphergenomics.org).^45^ *SRSF1* is also intolerant to missense variants (*Z* score = 3.96 according to gnomAD v.2.1.1).

**Table 1:** Molecular data of individuals with *SRSF1* variants

**Subjects**	**Genomic change**^g^	**Coding change**^h^	**Protein change**	**Variant type**	**Inheritance**	**Polyphen-2 score**	**Varsome prediction**^a^^,^^b^^,^^c^^,^^d^^,^^e^^,^^f^
I1	g.56083708_56083709del	c.377_378del	p.Ser126Trpfs^∗^17	Frameshift	Not inherited from the mother	–	Pathogenic: PVS1 PM2 PS2
I2, I3, I4	g.56083236C>T	c.478G>A	p.Val160Met	Missense	*De novo*	Probably damaging 0.999	Likely pathogenic: PS2 PM2 PP3
I5	g.56082937dup	c.579dup	p.Val194Serfs^∗^2	Frameshift	*De novo*	–	Pathogenic: PVS1 PM2 PS2
I6	g.55806534_56540597del	Not applicable	Not applicable	Deletion	*De novo*	–	–
I7	g.56084417G>A	c.82C>T	p.Arg28^∗^	Nonsense	*De novo*	–	Pathogenic: PVS1 PM2 PS2
I8	g.56083166T>C	c.548A>G	p.His183Arg	Missense	*De novo*	Probably damaging 1	Likely pathogenic: PS2 PM2 PP3
I9	g.56084380C>A	c.119G>T	p.Gly40Val	Missense	*De novo*	Probably damaging 1	Likely pathogenic: PS2 PM2 PP3
I10	g.56084402C>A	c.97G>T	p.Glu33^∗^	Nonsense	*De novo*	–	Pathogenic: PVS1 PM2 PS2
I11	g.56082914del	c.601del	p.Ser201Valfs^∗^87	Frameshift	*De novo*	–	Pathogenic: PVS1 PM2 PS2
I12	g.56083875C>T	c.208G>A	p.Ala70Thr	Missense	*De novo*	Probably damaging 1	Likely pathogenic: PS2 PM2 PP3
I13	g.56084428G>A	c.71C>T	p.Pro24Leu	Missense	*De novo*	Probably damaging 1	Likely pathogenic: PP3 PM2 PP2 PS2
I14	g.56083852A>C	c.231T>G	p.Tyr77^∗^	Nonsense	*De novo*	–	Pathogenic: PVS1 PM2 PS2
I15	g.56083832A>C	c.251T>G	p.Leu84Arg	Missense	*De novo*	Probably damaging 1	Likely pathogenic: PS2 PM2 PP3
I16	g.56084369C>T	c.130G>A	p.Asp44Asn	Missense	*De novo*	Benign 0.029	Uncertain significance: PP2 PM2 PS2 BP4
I17	g.55442363_56309063del	Not applicable	Not applicable	Deletion	*De novo*	–	–

a PVS1, predicted loss-of-function variant.

b PS2, *de novo* (both maternity and paternity confirmed) in an individual with the disease and no family history.

c PM2, absent from controls (or at extremely low frequency if recessive) in Exome Sequencing Project, 1000 Genomes Project, or Exome Aggregation Consortium.

d PP2, missense variant in a gene that has a low rate of benign missense variation and where missense variants are a common mechanism of disease.

e PP3, multiple lines of computational evidence support a deleterious effect on the gene or gene product.

f BP4, multiple lines of computational evidence suggest no impact on gene or gene product.

g GenBank: NC_000017.10

h GenBank: NM_006924.5

We performed a submission in the Matchmaker exchange tool GeneMatcher and the Undiagnosed Diseases Network International (UDNI) and contacted the referring physicians of 16 other individuals with rare *SRSF1* heterozygous variants (Figure 1A).^46^ A standardized questionnaire was sent to the referring physicians to collect molecular and clinical data including growth, neurodevelopment, congenital malformations, skeletal abnormalities, and facial features. The cohort was composed of 17 individuals, 10 females and 7 males, with DD, ID, hypotonia, behavioral disorders, and skeletal and cardiac anomalies as main features (Tables 2 and S1, see supplemental note), from 16 unrelated families (Figure 1B). Non-recurrent facial dysmorphic features were observed in many individuals (Figure 1C).

**Table 2:** Clinical characterization of individuals with heterozygous *SRSF1* variants

**Subjects**	**I1**	**I2**	**I3**	**I4**	**I5**	**I6**	**I7**	**I8**	**I9**	**I10**	**I11**	**I12**	**I13**	**I14**	**I15**	**I16**	**I17**
**Sex**	**F**	**F**	**M**	**M**	**F**	**F**	**M**	**F**	**M**	**M**	**F**	**F**	**F**	**M**	**M**	**F**	**F**
**Pregnancy/Birth**																	

Pregnancy complications	+	+	–	–	–	–	+	–	–	–	–	–	–	+	+	–	+
Gestational weeks	36	40	38	Full term	39	37	31	38	Full term	ND	Full term	41	39	41	39	38	ND
Birth weight (g) (SD)	1,700 (−2.6)	3,300 (−0.5)	3,180 (0)	2,920 (−1.5)	3,100 (−0.5)	2,550 (−0.6)	1,765 (+0.6)	2,790 (−0.6)	3,827 (+0.5)	ND	3,430 (0)	3,700 (+0.6)	3,997 (+1.7)	ND	2,980 (−1)	3,065 (+0.1)	ND
Birth length (cm) (SD)	39 (−3.6)	49.5 (−0.4)	51 (+1)	45.5 (−2.8)	63 (−0.2)	46 (−0.7)	40.5 (−0.2)	45 (−1.8)	53.5 (+1.3)	ND	ND	51 (+0.4)	50.8 (+0.9)	ND	50 (0)	51 (+1.3)	ND
Birth OFC (cm) (SD)	30.5 (−1.5)	35 (+0.4)	35 (+0.4)	35 (−0.1)	34 (−0.3)	32 (−1)	30 (+0.4)	33.5 (−0.2)	35 (−0.2)	ND	ND	34 (−0.5)	ND	ND	35 (+0.2)	34 (+0.2)	ND
Neonatal complications	+	–	+	+	–	+	–	–	+	–	+	–	+	+	–	+	+

**Growth**																	

Age at last visit	18 years	4 years 9 months	2 years	34 years	6 years 8 months	4 years	5 years 2 months	3 years 1 month	8 years 5 months	23 years 9 months	5 years	3 years 8 months	2 years 2 months	28 years	18 years	1 year 2 months	13 years 6 months
Weight last visit (kg) (SD)	54.2 (−0.3)	17.5 (+0.2)	11.6 (−0.8)	65.6 (−0.4)	20 (−0.5)	14.5 (−0.8)	19 (+0.1)	10 (−2.8)	128 (+0.1)	41 (−3.3)	19.7 (+0.5)	17.8 (+1)	12.7 (+0.2)	57.7 (−1.4)	57.6 (−1)	8.9 (−1.2)	ND
Height last visit (cm) (SD)	152 (−1.8)	103 (−0.5)	86 (−0.4)	189.5 (+2)	119.5 (+0.1)	98.3 (−0.6)	112.8 (+0.7)	84 (−2.7)	28 (+0.4)	156 (−2.9)	109 (+0.3)	99 (+0.7)	84.2 (+0.9)	174 (−0.4)	180.5 (+0.7)	77.4 (+0.5)	144 (−2)
BMI last visit (kg/m^2^)	23.5 (+0.6)	16.5 (+0.8)	15.7 (−0.2)	18.3 (−1.6)	14 (−0.9)	15 (−0.2)	14.9 (−0.3)	14.2 (−1)	17.1 (+0.7)	16.8 (−2.4)	16.6 (+0.8)	18.2 (+1.8)	17.9 (+1.6)	19.1 (−0.9)	17.7 (−1.4)	14.9 (−0.9)	ND
OFC last visit (cm) (SD)	55 (+0.6)	51 (+0.6)	50 (+1)	59 (+2.7)	52.5 (+0.6)	48.5 (−0.7)	51 (−0.1)	45.5 (−2.9)	51.8 (−0.4)	54.5 (−0.4)	48.7 (−1.1)	49 (−1)	47.8 (+0.1)	56 (+0.6)	55 (−0.1)	47 (+1.1)	50 (−2.6)
Failure to thrive	+	–	–	–	–	ND	–	+	–	+	–	+	–	+	–	–	+
Truncal overweight	+	–	Trunk adiposity	–	–	–	–	–	–	–	–	–	+	–	–	–	–

**Neurological abnormalities**																	

ID/DD	Mild to moderate	Mild to moderate	Mild to moderate	LD	Mild	Yes	Severe	Severe	Borderline	Severe	Moderate	Moderate	Yes	Mild	Mild	Yes	Yes
Motor delay	+	+	+	–	+	+	+	+	–	+	–	+	+	+	–	+	+
Speech delay	+	+	+	+	+	+	+	+	+	+	–	+	+	+	+	+	+
Behavioral disorders	+	+	–	+	+	–	+	–	+	+	+	+	–	+	+	+	+
Hypotonia	+	+	+	+	Buccal hypotonia	–	–	+	–	+	–	–	+	+	+	+	ND
Seizures	–	–	–	–	–	–	–	–	–	+	–	–	–	+	–	+	–
Brain abnormalities MRI	+	+	–	–	–	ND	+	+	–	–	–	–	+	ND	ND	+	ND

**Neurosensory abnormalities**																	

Hearing loss	–	–	–	–	–	ND	–	–	+	–	–	–	–	–	–	–	–
Vision problems	+	+	–	ND	–	+	–	+	–	+	–	ND	+	–	+	–	+

**Congenital Malformations**																	

Cardiac	+	+	–	–	–	+	–	ND	+	+	–	–	–	+	–	–	ND
Urogenital/kidney	–	–	+	+	–	ND	–	–	+	+	–	ND	–	–	+	–	ND
Others	Polycystic spleen	–	–	–	–	–	–	–	–	–	–	–	–	Hernia, diastasis	–	–	–

**Skeletal abnormalities**																	

Scoliosis	+	–	+	+	–	–	–	–	–	–	–	–	–	+	+	–	–
Pectus deformity	–	–	–	+	–	+	–	–	–	–	–	–	–	+	+	–	–
Other	Genu varum, Equinova-rus	–	–	Arachno-dactyly	Brachymetatarsia Fallen arches	–	–	–	L1 vertebral hypoplasia Kyphosis	Asymme-tric chest	–	–	–	Dolichostenomelia Equinova-rus Postaxial hexa-dactyly	Metatarsus varus Arachnodactyly	–	Genu valgum

**Others**																	

Facial features	+	+	+	+	+	+	+	–	+	+	+	+	+	+	+	+	+
Cutaneous abnormalities	+	–	–	–	+	+	–	–	–	–	–	–	+	–	–	–	–
Other diagnosis	–	–	–	–	–	–	–	–	–	Diagnosed Hermansky Pudlak Syndrome	–	MBD6 variant	–	–	–	–	15q11.2 BP1-BP2 microdeletion

ND, no data; OFC, occipito-frontal circumference; BMI, body mass index.

In total, we observed 15 different *SRSF1* variants in 17 NDD individuals, including two microdeletions of less than one megabase and three frameshift, three nonsense, and seven missense variants (Table 1). All LoF variants were predicted to induce NMD, except variants c.579dup (p.Val194Serfs^∗^2) and c.601del (p.Ser201Valfs^∗^87) (Table 1), both located in the last exon (exon 4).^47^ Among the missense variants, 5 affected the RRM1 domain and 2 the RRM2 domain (Figure 1A). Individual 6 (F5-II-1) and individual 17 (F16-II-1) presented *de novo* heterozygous deletions of 734 kb and 866 kb, respectively, in the 17q22 region encompassing *SRSF1* (Table 1, Figure S1). The 15 SNV/indel alleles and the CNV deletions were absent from the gnomAD population database (gnomAD v.2.1.1, https://gnomad.broadinstitute.org) and confirmed to be *de novo* (Figure 1B) except for individual 1 (F1-II-1), for whom the variant was found not to be inherited from her mother, but the paternal sample was unavailable for testing. Individual 2 (F2-II-2) and individual 3 (F2-II-3) are siblings (Figure 1B), and they both have the same *SRSF1* variant (Table 1), suggesting germinal mosaicism in one parent. In three individuals, 10 (F9-II-3), 12 (F11-II-2), and 17 (F16-II-1), genetic analyses led to the identification of multilocus disease-causing or candidate genomic variations, i.e., genomic variations at more than one genetic locus accounting for distinct or blending phenotypes.^48^^,^^49^ Individual 10 (F9-II-3) also presented Hermansky-Pudlak syndrome 1 (MIM: 203300) due to a homozygous pathogenic variant in *HPS1* (MIM: 604982, c.973_974insC [GenBank: NM_000195.3] [p.Met325Thrfs^∗^128]). Individual 12 (F11-II-2) also harbors a *de novo* frameshift VUS in *MBD6* (MIM: 619458, c.2337dup [GenBank: NM_052897.4] [p.Gly780Trpfs^∗^13]); this gene is suspected to be associated with autism and language delay and could contribute to her phenotype.^50^ Individual 17 (F16-II-1) also has a 15q11.2 BP1-BP2 microdeletion, which can be associated with developmental and language delay, neurobehavioral disturbances, and psychiatric problems.^51^

### *In silico* functional prediction and structural modeling of SRSF1 missense variants

**Figure 2:**
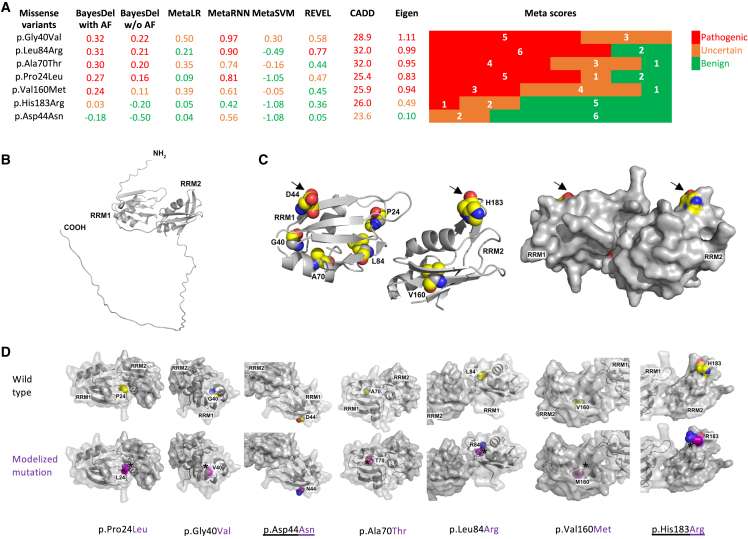
Bio-informatic pathogenicity predictions and structural modeling of missense variants (A) Pathogenicity prediction scores for the seven SRSF1 missense variants generated by eight meta-prediction tools (BayesDel with AF, BayesDel without AF, MetaLR, MetaRNN, MetaSVM, REVEL, CADD, Eigen) as implemented in the VarSome human genomic search engine.^52^ Colored bars on the right represent the number of meta-tools supporting pathogenic (red), uncertain (brown), and benign (green) predictions. (B) Structural prediction of SRSF1 using AlphaFold and PyMol. (C) Left: missense variants superimposed on the structural prediction. Right: Surface rendering of the SRSF1 protein structure. Arrows indicate p.Asp44Asn and p.His183Arg, two residues closer to the protein surface. (D) Structural prediction of the seven missense variants. The underlined variants, p.Asp44Asn and p.His183Arg, are located at the protein surface. The other variants are more oriented toward the internal structure of the protein. Carbons are represented in yellow, nitrogens in blue, and oxygens in red for the wild-type amino acids; carbons are represented in purple in the modelized alterations. Asterisks indicate potential steric clashes in the mutated structures.

The identification of nonsense, frameshift, and deletion variants suggested that haploinsufficiency of *SRSF1* is the most likely common pathogenic mechanism in SRSF1-related NDD; therefore, we hypothesized that pathogenic missense variants likely also behave as LoF alleles. Functional *in silico* and *in vivo* evidence supporting LoF was thus essential to classify these missense variants as being (likely) pathogenic. We therefore first used eight *in silico* meta-predictors (BayesDel with AF, BayesDel without AF, MetaLR, MetaRNN, MetaSVM, REVEL, Eigen, CADD) embedded in the human genomic search engine VarSome to obtain functional prediction scores of the seven missense variants (Figure 2A).^52^ Recent studies indicate that BayesDel outperforms most other meta-predictors for clinical missense variant classification.^53^^,^^54^ Although the eight tools were not unanimous for any of the seven missense variants, c.548A>G (p.His183Arg) and c.130G>A (p.Asp44Asn) (Table 1) obtained lower prediction scores with most meta-predictors, suggesting a reduced pathogenic potential and perhaps hypomorphic nature for these two variants. Higher scores were obtained for the five other missense variants, c.119G>T (p.Gly40Val), c.251T>G (p.Leu84Arg), c.208G>A (p.Ala70Thr), c.71C>T (p.Pro24Leu), and c.478G>A (p.Val160Met) (Table 1), with BayesDel supporting potential pathogenicity for all five.

Next, we obtained the human SRSF1 protein structure from the AlphaFold Protein Structure Database. This showed that both RRMs of SRSF1 are brought in close vicinity by the tertiary structure (Figure 2B). We then modeled the seven missense variants, which were all located in the RRM domains: five in the RRM1 and the last two in the RRM2 domain. A surface rendering of both RRMs showed that residues affected by the missense variants are in close interaction with each other, with positions Pro24 and Leu84 possibly involved in establishing or maintaining the interaction between the two RRMs. In addition, the surface view showed that most of the mutated amino acids are located inside the protein structure, which is more in favor of internal misfolding than with altered interactions with partners or other proteins (Figures 2C and 2D). Interestingly, the only positions that are pointed slightly out of the surface of the RRMs are Asp44 and His183, possibly explaining the reduced predicted pathogenic potential of p.Asp44Asn and p.His183Arg (Figure 2D).

### *In vivo* modeling in *Drosophila* identifies SRSF1 splicing-defective clinical variants

Since the *in silico* predictions of the seven suspected disease-causing *de novo* missense variants displayed diverging levels of support for pathogenicity (Figure 2), and two LoF variants (p.Val194Serfs^∗^2 and p.Ser201Valfr^∗^87) were predicted to escape NMD, we decided to use a quantitative *Drosophila* SRSF1 splicing model to further address the pathogenicity of missense and truncating variants (see supplemental information for additional details, Figures S2–S4).

The visual system of flies is studied extensively, and many of the signaling pathways involved in its development have been identified.^55^^,^^56^ The compound eye of *Drosophila* consists of more than 700 hexagonal ommatidia. Each ommatidium contains eight light-sensing neuronal photoreceptor cells and 12 supporting non-neuronal cells (cone and pigment cells). The eye therefore serves as a powerful genetic model system for studying nervous system development.^56^

**Figure 3:**
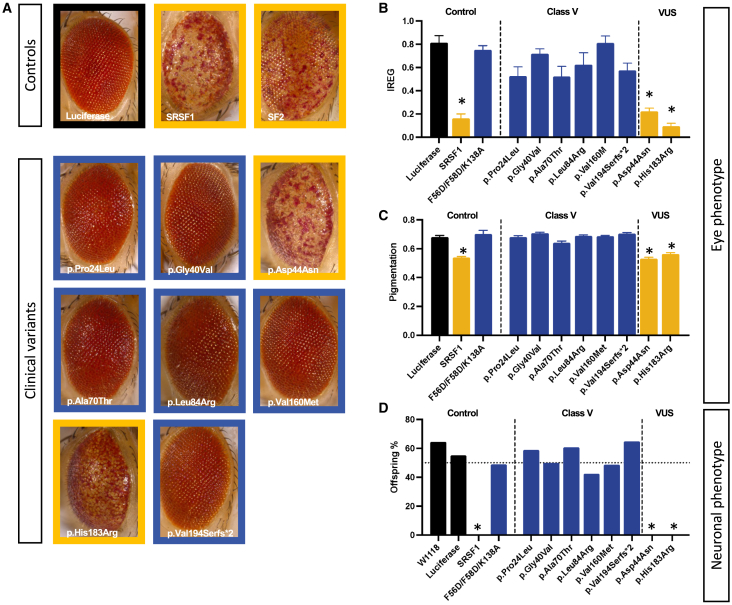
Eye and neuronal splicing read-outs of clinical variants (A) Representative eye picture of flies expressing luciferase, SF2, SRSF1, and SRSF1 clinical variants in the fly eye under the control of the GMR-GAL4 enhancer. (B) The irregularity score or regularity index of flies expressing luciferase (negative control), SRSF1 (positive control), a SRSF1 splicing-deficient protein (F56D/F58D/K138A) (negative control), and SRSF1 clinical variants. n > 10, ^∗^p < 0.01, data are represented as mean ± SEM. p.Asp44Asn and p.His183Arg display a lower IREG score similar to the SRSF1-overexpressing flies. (C) Pigmentation score measuring the depigmentation in the different controls and the clinical variants. n > 10, ^∗^p < 0.01, data are represented as mean ± SEM. (D) Offspring frequencies were measured in flies expressing luciferase, SRSF1, a splicing-deficient SRSF1 protein, and the clinical variants pan-neuronally. n > 10, ^∗^p < 0.01.

All identified SRSF1 residues affected by missense variants (except for p.Asp44Asn) are conserved in the *Drosophila* ortholog SF2 (FlyBase Gene Report: Dmel∖SF2) (Figure 1A). In the literature, overexpression of WT SF2 in flies leads to phenotypic alterations in eye organogenesis, including quantitative changes such as depigmentation and loss of eye regularity, due to alternative splicing of key genes involved in eye development.^57^ We replicated these results and found that eye-specific overexpression of splicing-active versions of SF2 and SRSF1 indeed led to an eye phenotype, whereas a previously described splicing-inactive version of SRSF1 (referred to here as SRSF1 F56D/F58D/K138A) lost this capacity (Figure S2, supplemental information).^24^^,^^25^ Both eye roughness (IREG score, Figures 3A and 3B) and depigmentation (Figures 3A–3C) were quantified and used to estimate the phenotype-inducing capacity, and hence the splicing activity, of the clinical variants. Variants p.Pro24Leu, p.Gly40Val, p.Ala70Thr, p.Leu84Arg, p.Val160Met, and p.Val194Serfs^∗^2 resulted in the loss of the phenotype induced by SRSF1 overexpression. The IREG and pigmentation scores were comparable to WT and splicing-deficient SRSF1 F56D/F58D/K138A eyes, hence different from the active protein. These data indicate that these variants behaved as “loss of splicing activity” variants. Variants p.Asp44Asn and the p.His183Arg were as potent as the WT protein to induce the phenotype and thus did not show a loss of the splicing activity. We excluded that tissue-specific splicing alterations might explain these findings (Figure 3D). All variants that were unable to induce an eye phenotype also failed to induce pharate adult lethality upon overexpression in the nervous system, whereas p.Asp44Asn and p.His183Arg were lethal and not different from WT. Furthermore, WT SRSF1, as well as p.His183Arg and p.Val160Met, all localized to the nuclear compartment in CCAP neurons as expected, suggesting that altered subcellular localization is likely not responsible for the varying results in the *in vivo* splicing assay among missense variants (Figure S4). Taken together, our results show that LoF, truncating variants abolishing the R/S domain, and 5 out of 7 missense variants display strongly reduced splicing activity, in line with haploinsufficiency as the underlying genetic mechanism in SRSF1-mediated NDD. The analysis is in accordance with the *in silico* predictions of a reduced pathogenic potential for p.Asp44Asn and p.His183Arg.

### Episignature analysis

To determine whether *SRSF1* variants would cause a detectable change in DNA methylation, we compared methylation beta values between nine samples with confirmed *SRSF1* splicing-defective pathogenic variants (i.e., individuals I1–I6, I11, I14, and I15) against matched controls. We identified 107 differentially methylated CpG probes for the *SRSF1* episignature (Table S2). Unsupervised clustering methods, including hierarchical (heatmap) and MDS, demonstrated that the CpG probes selected as a clinical biomarker were capable of segregating the *SRSF1* samples with confirmed pathogenic variants from controls (Figures S5A and S5B). “Leave one out” cross-validation was performed, and the results were visualized using unsupervised heatmap and MDS clustering methods, which confirmed the robustness and sensitivity of the episignature (Figure S6). All testing samples were correctly clustered with the discovery training samples (Figure S5). A SVM model was constructed using the 107 selected episignature probes. All *SRSF1* samples with confirmed pathogenic variants showed a methylation variant pathogenicity (MVP) score close to 1, indicating the similarity of the observed methylation pattern to the *SRSF1* episignature (Figure S5C).

**Figure 4:**
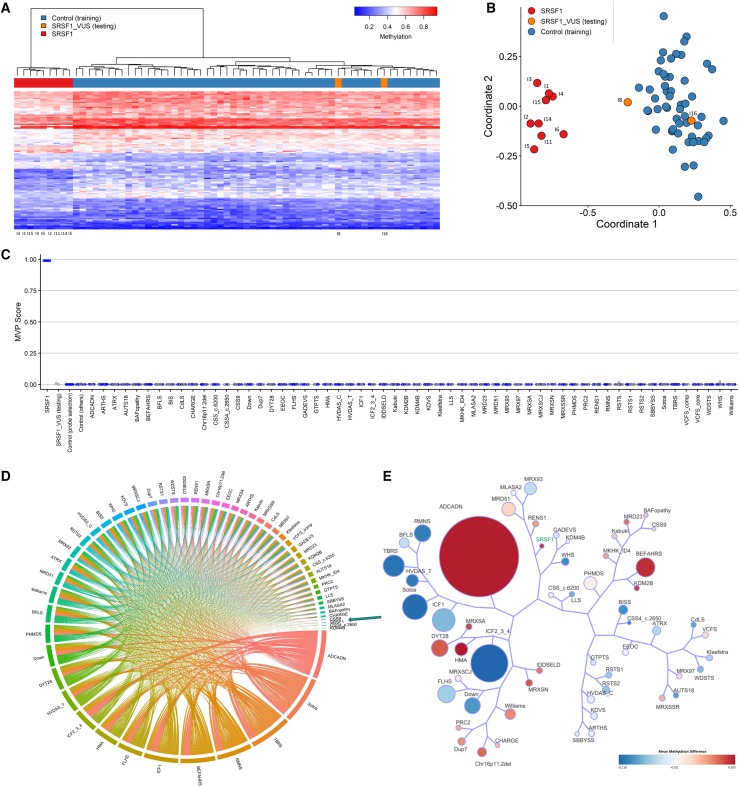
Episignature assessment of *SRSF1* VUSs p.Asp44Asn and p.His183Arg (A) Heatmap indicates that the two VUS samples (orange) are clustering with controls (blue) and away from the *SRSF1* samples with confirmed pathogenic variants (individuals I1–I6, I11, I14, and I15 used for episignature discovery) (red). Each row represents one of the 107 probes selected as the episignature, and each column represents an individual with either an *SRSF1* variant (red or orange) or a control (blue). (B) Multidimensional scaling plot (MDS) also shows clustering of the *SRSF1* VUS samples with controls. (C) Support vector machine classifier model (SVM) shows that the VUSs have a probability score (methylation variant pathogenicity score, MVP) of close to 0 compared with the *SRSF1* samples carrying confirmed pathogenic variants with MVP scores of close to 1. The model is trained using the 107 selected SRSF1 episignature probes and 75% of controls and other neurodevelopmental disorder samples on EpiSign (blue circles). The 25% remaining are used as testing samples (gray circles). (D) Circos plot representing the differentially methylated probes (DMPs) shared between each pair of cohorts. The thickness of the connecting lines indicates the number of probes shared between the paired cohorts. SRSF1 cohort is indicated by the green arrow. (E) Tree-and-leaf visualization of Euclidean clustering of the *SRSF1* cohort alongside the 56 other EpiSign disorders using the top n DMPs for each cohort, where n = 500 or the max number of DMPs available if <500. Cohort samples are aggregated using the median value of each probe within a group. Each leaf (node) represents a cohort, with node sizes illustrating relative scales of the number of selected DMPs for the corresponding cohort, and node colors indicative of the global mean methylation difference where blue is more hypomethylated and red hypermethylated. The *SRSF1* cohort with confirmed pathogenic variants is highlighted in green. ADCADN, cerebellar ataxia deafness and narcolepsy syndrome; AUTS18, susceptibility to autism 18; BEFAHRS, Beck-Fahrner syndrome; BFLS, Borjeson-Forssman-Lehmann syndrome; BISS, blepharophimosis intellectual disability SMARCA2 syndrome; CdLS, Cornelia de Lange syndrome; CHARGE, CHARGE syndrome; Chr16p11.2del, chromosome 16p11.2 deletion syndrome; CSS, Coffin-Siris syndrome; CSS4, Coffin-Siris syndrome 4; CSS9, Coffin-Siris syndrome 9; Down, Down syndrome; Dup7, 7q11.23 duplication syndrome; DYT28, dystonia 28; EEOC, epileptic encephalopathy-childhood onset; FLHS, Floating-Harbor syndrome; GTPTS, genitopatellar syndrome; HMA, Hunter McAlpine craniosynostosis syndrome; HVDAS, Helsmoortel-van der Aa syndrome; ICF, immunodeficiency-centromeric instability-facial anomalies syndrome; IDDSELD, intellectual developmental disorder with seizures and language delay; Kabuki, Kabuki syndrome; KDVS, Koolen-De Vries syndrome; Kleefstra, Kleefstra syndrome; LLS, Luscan-Lumish syndrome; MKHK, Menke-Hennekam syndrome; MLASA2, myopathy lactic acidosis and sideroblastic anemia 2; MRD23, intellectual developmental disorder 23; MRD51, intellectual developmental disorder 51; MRX93, intellectual developmental disorder X-linked 93; MRX97, intellectual developmental disorder X-linked 97; MRXSA, intellectual developmental disorder X-linked syndromic Armfield type; MRXSCH, intellectual developmental disorder X-linked syndromic Christianson type; MRXSCJ, intellectual developmental disorder X-linked syndromic Claes-Jensen type; MRXSN, intellectual developmental disorder X-linked syndromic Nascimento type; MRXSSR, intellectual developmental disorder X-linked syndromic Snyder-Robinson type; PHMDS, Phelan-McDermid syndrome; PRC2, PRC2 complex (Weaver and Cohen-Gibson) syndrome; RENS1, Renpenning syndrome; RMNS, Rahman syndrome; RSTS, Rubinstein-Taybi syndrome; SBBYSS, Ohdo syndrome; Sotos, Sotos syndrome; TBRS, Tatton-Brown-Rahman syndrome; WDSTS, Wiedemann-Steiner syndrome; WHS, Wolf-Hirschhorn syndrome; Williams, Williams syndrome.

Previous studies have shown that episignatures are clinical biomarkers that can be used to aid in the classification of VUSs and screening of individuals with suspected genetic disorders.^58^^,^^59^ Using the 107 selected episignature probes, we assessed the two samples with normal splicing activity in our *Drosophila* assay, p.Asp44Asn and p.His183Arg, and classified these samples using unsupervised (hierarchical and MDS) clustering as well as supervised SVM methods. Both samples clustered with controls in heatmap and MDS (Figures 4A and 4B) and had an MVP prediction score of close to 0 (Figure 4C). These results show that p.Asp44Asn and p.His183Arg did not exhibit an aberrant DNA methylation pattern in common with the mapped *SRSF1* episignature, confirming the *in silico* predictions and *in vivo* results obtained in *Drosophila*.

### Functional correlation of the *SRSF1* genome-wide methylation profile to other EpiSign V3 classifier disorders

To perform functional correlation analyses, we compared the *SRSF1* cohort to episignature-negative age- and sex-matched controls using probes present on both the Illumina EPIC and 450K arrays. Probes with a methylation difference >5% and adjusted p value <0.01 were retained and resulted in a list of 1,485 DMPs (Table S3). The *SRSF1* DMPs were compared to the DMPs of 56 other EpiSign disorders previously described.^37^ Heatmap showed the percentage of the DMPs shared between cohorts; the highest overlaps for the *SRSF1* DMPs were with autosomal dominant cerebellar ataxia, deafness, and narcolepsy (ADCADN; MIM: 604121) (∼49%), Hunter McAlpine syndrome (HMA; MIM: 601379) (∼9%), Tatton-Brown-Rahman syndrome (TBRS; MIM: 615879) (∼10%), Sotos syndrome (Sotos; MIM: 117550) (∼11%), and Rahman syndrome (RMNS; MIM: 617537) (∼11%) (Figure S5D). The overlap with ADCADN is likely the result of the sheer number of DMPs contained within the ADCADN methylation profile (n = 151,848). These overlaps were further visualized in a circos plot (Figure 4D). These overlaps in DMPs may indicate a common underlying biological process shared between these disorders and may provide insights into the molecular pathways of these conditions.

Using the DMRcate algorithm with p-cutoff set to default (FDR) and beta-cutoff input of 0.05 mean methylation difference and 5 CpGs within 1,000 bp, we identified 34 DMRs (Table S4).^43^ Thirteen DMRs were hypomethylation events and 21 hypermethylation. Next, we annotated the genomic locations of the DMPs and the DMRs in relation to CpG islands and genes. This showed that the DMPs are predominantly found in the CpG shores and in promoter regions (Figure S7). Annotation was also performed for DMRs in relation to CpG islands and genes and showed the DMPs predominantly in CpG islands and a pronounced association with promoter regions when annotated in relation to genes. Next, all DMPs were used to calculate the mean beta values for each cohort and determine the overall methylation trend, i.e., hypo- or hypermethylation (Figure S8). Genome-wide methylation profile of the *SRSF1* cohort showed an overall hypermethylation trend, in line with the majority of hypermethylation DMRs identified. Lastly, we aimed to analyze the relatedness of genome-wide methylation profiles by comparing the *SRSF1* cohort and all 56 other disorders. To assess this relationship, clustering analysis was performed using up to the top 500 DMPs for each cohort. For cohorts with less than 500 DMPs, the total DMPs for those cohorts were used in the analysis. Results were visualized using a binary tree with each node representative of a cohort (Figure 4E). *SRSF1* is shown to cluster closest to Renpenning syndrome 1 (RENS1; MIM: 309500) in the tree-and-leaf plot, and both show a global hypermethylation profile.

### ACMG classification of the identified clinical *SRSF1* variants

Our functional studies were important to establish *SRSF1* haploinsufficiency as the common genetic mechanism in SRSF1-related NDD, also in individuals with missense variants. To examine the exact impact of functional studies on the ACMG variant classification, we compared the ACMG scores and classifications before and after functional analysis (including variant modeling in *Drosophila* and/or epigenetic analysis). For 9 out of 11 variants for which functional data were available (Table 3), the total scores increased in such a way that it resulted in a reclassification from “likely pathogenic” to “pathogenic” in six (6/9, 55%). For one variant the total score further decreased, resulting in a reclassification from “likely pathogenic” to “uncertain significance” for p.His183Arg, while p.Asp44Asn remained a variant of “uncertain significance” after functional analysis. In total, from the 13 intragenic *SRSF1* variants, ACMG criteria classified 11 as “pathogenic” (85%) and 2 as “uncertain” (15%). The frequencies of the main clinical features described in individuals harboring *SRSF1* variants before and after reclassification are summarized in Table 4. These results highlight the clinical importance of functional studies for variant interpretation.

**Table 3:** ACMG classification of *SRSF1* clinical variants before and after functional studies

**SRSF1 variant**	**Splicing deficient (*Drosophila* assay)**	**SRSF1 episignature**	**ACMG classification (before functional studies)**	**Total score**	**ACMG classification (after functional studies)**	**Total score**
p.Ser126Trpfs^∗^17	NA	Yes	Likely Pathogenic: PVS1, PM2	9	Pathogenic: PVS1, PM2, PS3	13
p.Val160Met	Yes	Yes	Likely Pathogenic: PP3, PM2, PP2, PS2	8	Pathogenic: PP3, PM2, PP2, PS2, PS3	12
p.Val194Serfs^∗^2	Yes	Yes	Pathogenic: PVS1, PM2, PS2	13	Pathogenic: PVS1, PM2, PS2, PS3	17
p.Arg28^∗^	NA	NA	Pathogenic: PVS1, PM2, PS2	13	NA	NA
p.His183Arg	No	No	Likely Pathogenic: PM2, PP2, PS2	6	Uncertain significance: PM2, PP2, PS2, BS3	2
p.Gly40Val	Yes	NA	Likely Pathogenic: PP3, PM2, PP2, PS2	8	Pathogenic: PP3, PM2, PP2, PS2, PS3	12
p.Glu33^∗^	NA	NA	Pathogenic: PVS1, PM2, PS2	13	NA	NA
p.Ser201Valfs^∗^87	Yes	Yes	Pathogenic: PVS1, PM2, PS2	13	Pathogenic: PVS1, PM2, PS2, PS3	17
p.Ala70Thr	Yes	NA	Likely Pathogenic: PP3, PM2, PP2, PS2	8	Pathogenic: PP3, PM2, PP2, PS2, PS3	12
p.Pro24Leu	Yes	NA	Likely Pathogenic: PP3, PM2, PP2, PS2	8	Pathogenic: PP3, PM2, PP2, PS2, PS3	12
p.Tyr77^∗^	NA	Yes	Pathogenic: PVS1, PM2, PS2	13	Pathogenic: PVS1, PM2, PS2, PS3	17
p.Leu84Arg	Yes	Yes	Likely Pathogenic: PP3, PM2, PP2, PS2	8	Pathogenic: PP3, PM2, PP2, PS2, PS3	12
p.Asp44Asn	No	No	Uncertain significance: PM2, PP2, PS2, BP4	4	Uncertain significance: PM2, PP2, PS2, BP4, BS3	0

ACMG variant classification using Varsome: PVS1 (very strong, +8 points): null variant (nonsense, frameshift, canonical +/−1 or 2 splice sites, initiation codon, single or multiexon deletion) in a gene where LoF is a known mechanism. PM2 (supporting, +1 point): absent from controls (or at extremely low frequency if recessive) (based on gnomAD frequencies). PP2 (supporting, +1 point): missense variant in a gene that has a low rate of benign missense variation and in which missense variants are a common mechanism of disease (based on gnomAD missense *Z* score). PP3 (moderate, +2 points): multiple lines of computational evidence support a deleterious effect on the gene or gene product (based on BayesDel_addAF score). PS2 (strong, +4 points): *de novo* (both maternity and paternity confirmed) in an individual with the disease and no family history. PS3 (strong, +4 points): well-established *in vitro* or *in vivo* functional studies show no damaging effect on protein function or splicing. BS3 (strong, −4 points): well-established *in vitro* or *in vivo* functional studies show no damaging effect on protein function or splicing. BP4 (moderate, −2 points): multiple lines of computational evidence suggest no impact on gene or gene product (based on BayesDel_addAF score).

**Table 4:** Summary of main clinical features described in individuals harboring *SRSF1* variants

	**Number of individuals with pathogenic *SRSF1* variant**	**Total number of individuals with *SRSF1* variant including VUS variants**
ID or DD	15/15	17/17
Speech delay	14/15	16/17
Motor delay	11/15	13/17
Hypotonia	9/14	11/16
Behavior disorders	12/15	13/17
Abnormal brain MRI	4/10	6/12
Cardiac malformation	6/13	6/14
Urogenital malformation	6/11	6/13
Skeletal abnormalities	10/15	10/17
Marfanoid features	3/15	3/17

DD, developmental delay; ID, intellectual disability; VUS, variant of uncertain significance.

## Discussion

In this study, we clinically and molecularly described a cohort of 17 individuals from 16 families, with 15 different heterozygous germline variants in *SRSF1*. The main clinical features were DD or ID. Other features were variably present and included skeletal anomalies, behavioral disorders, congenital heart defects, and urogenital malformation. Among the five adult individuals, three presented marfanoid features with long and thin habitus, pectus excavatum or carinatum, dolichostenomelia, arachnodactyly, scoliosis, and highly arched palate. In the literature, phenotypes linked with SRSF1 overexpression causing dysregulation of alternative splicing have been associated with cancer.^30^^,^^31^^,^^32^ Here, we provide genetic, epigenetic, and structural arguments, in combination with evidence gathered from *in vivo* functional modeling, for a LoF as a pathogenic mechanism. The identification of microdeletions encompassing *SRSF1*, nonsense, and frameshift variants points toward haploinsufficiency. This is further supported by gnomAD data showing that *SRSF1* is highly intolerant to LoF, according to the pLI = 0.98 and LOEUF = 0.24 scores. For five of the seven missense variants, p.Gly40Val, p.Leu84Arg, p.Ala70Thr, p.Pro24Leu, and p.Val160Met, we obtained combined *in silico* evidence, *in vivo* modeling arguments, and supportive epigenetic data to classify them as pathogenic LoF variants. In contrast, for the two missense variants, p.Asp44Asn and p.His183Arg, the data did not support their pathogenic role. The functional and structural prediction tools that were used point toward a lower pathogenicity for the latter two variants. Structurally, these variants were located at the protein surface, whereas the other five missense variants are predicted to cause internal misfolding. These *in silico* data were consistent with the data obtained in two independent functional splicing read-outs in our *Drosophila* model system. Both in the fly eye and in the nervous system, overexpression of SRSF1 and its *Drosophila* ortholog, SF2, leads to severe phenotypes due to splicing alterations. All five missense variants lost their potency to induce eye and brain phenotypes, pointing toward LoF mutations, whereas p.Asp44Asn and p.His183Arg retained this ability. Interestingly, p.Asp44Asn was the only missense variant located in a less conserved region of the RRM1 domain and not conserved in the fruit fly. We validated the use of *Drosophila* to model SRSF1 function by providing evidence for structural and functional conservation even at the molecular level. Our data are in line with previously reported experimental studies in *Drosophila* showing that the splicing activity of *SRSF1* is evolutionarily conserved.^25^^,^^57^ A combination of biochemically characterized splicing variants and transcriptome analysis make us hypothesize that we are mainly modeling splicing alteration involving U1 snRNP activity. As SRSF1 is shown to have other splicing functions apart from U1 snRNP activity, this might be a second explanation for the absence of phenotype-inducing capacity of both modeled variants. Thirdly, both variants might exert their toxicity through a different pathogenic mechanism. Both variants are indeed located at the protein surface and therefore more likely to intervene with protein interaction and might hamper other functions of SRSF1. To gain insight into the possible existence of a common disease pathway, we investigated the epigenetic signature associated with *SRSF1* variants in blood obtained from the affected individuals. DNA methylation data also corroborated the data obtained in *Drosophila*. Therefore, we argue that these variants be classified as being of uncertain significance (i.e., VUS). However, it is important to highlight that they should not be classified as benign because the functional models used in this study do not address all of the functions of this protein. Further allelic series studies and genome analyses in affected cohorts may clarify whether these variants are pathogenic or benign.

Besides the identification of *SRSF1* haploinsufficiency as a cause of ID/DD, we possibly identified *SRSF1* as being an important gene responsible for the neurodevelopmental features associated with 17q22 microdeletions as well. Individuals with 1.8–2.5 Mb microdeletions of the 17q22 region have been reported in the literature.^60^^,^^61^^,^^62^^,^^63^^,^^64^ An important causal gene related to the clinical manifestations of 17q22 microdeletion is *NOG* (MIM: 602991). When it is included in the microdeletion, *NOG*-related bone and joint features such as symphalangism, conductive hearing loss, and joint contractures are present, as are visual impairment and facial dysmorphic features.^60^ However, additional features not related to *NOG* haploinsufficiency may also be present such as ID and ADHD.^60^ Among the reported individuals with 17q22 contiguous microdeletions, six had loss of *SRSF1* and presented with syndromic ID.^60^^,^^61^^,^^62^^,^^63^ More recently, Pang et al. reported a family with 1.6 Mb microdeletion in chromosome 17q22 with *NOG*-related symphalangism spectrum disorder including conductive hearing loss, proximal symphalangism of the fifth fingers, small palpebral fissures, broadened hemicylindrical nose with a bulbous tip, amblyopia, and strabismus without ID or any other neurodevelopmental abnormalities.^64^ In comparison with the genes included in the microdeletion of their family and the genomic intervals deleted by other microdeletions in chromosome 17q22, the authors suggested two candidate genomic intervals for ID. Among the distal candidate genomic intervals, *SRSF1* was included.

In our cohort, we reported two microdeletions of the 17q22 region with sizes of 734 kb and 866 kb, including *SRSF1* but excluding *NOG*. Therefore, our study further supports the role of *SRSF1* as one of the critical “driver genes” for the 17q22 contiguous microdeletion-related syndrome, accounting for at least part of the neurodevelopmental features associated with it. Interestingly, the DNA methylation analysis showed that the sample with the CNV variant from individual 6 (F5-II-1) clustered with the other samples with pathogenic missense, frameshift, or nonsense variants in *SRSF1*, supporting the growing evidence that intragenic variants within critical genes and microdeletions encompassing them share similar DNA methylation profiles.^65^^,^^66^^,^^67^

In conclusion, we described a cohort of individuals with heterozygous variants in *SRSF1*, responsible for a syndromic form of DD characterized by learning disabilities with mild to severe ID and, to a variable extent, associated with skeletal anomalies and with cardiac or urogenital malformations. Additional functional studies are needed to fully understand the pathogenic mechanisms at play in the *SRSF1*-related NDD.

## Data Availability

The published article includes all datasets generated or analyzed during this study. *SRSF1* genetic variants identified in our study were submitted to ClinVar (https://www.ncbi.nlm.nih.gov/clinvar/) under the accession IDs ClinVar: SCV003803742–SCV003803756.

## Web resources

ClinVar, https://www.ncbi.nlm.nih.gov/clinvar/
Decipher, https://www.deciphergenomics.org
GeneMatcher, https://www.genematcher.org
gnomAD, https://gnomad.broadinstitute.org
gplots, https://CRAN.R-project.org/package=gplots
PolyPhen-2, http://genetics.bwh.harvard.edu/pph2/
Varsome, https://varsome.com/

## Supplemental information

Document S1. Figures S1–S8, Table S1, supplemental note, and supplemental methods

Data S1. Tables S2–S4

Document S2. Article plus supplemental information
